# κ-Opioid Receptor Stimulation Improves Endothelial Function via Akt-stimulated NO Production in Hyperlipidemic Rats

**DOI:** 10.1038/srep26807

**Published:** 2016-05-26

**Authors:** Fei Tian, Xu-Yang Zheng, Juan Li, Shu-Miao Zhang, Na Feng, Hai-Tao Guo, Min Jia, Yue-Min Wang, Rong Fan, Jian-Ming Pei

**Affiliations:** 1 Department of Physiology, National Key Discipline of Cell Biology, Fourth Military Medical University, Xi’an 710032, China

## Abstract

This study was designed to investigate the effect of U50,488H (a selective κ-opioid receptor agonist) on endothelial function impaired by hyperlipidemia and to determine the role of Akt-stimulated NO production in it. Hyperlipidemic model was established by feeding rats with a high-fat diet for 14 weeks. U50,488H and nor-BNI (a selective κ-opioid receptor antagonist) were administered intraperitoneally. *In vitro*, the involvement of the PI3K/Akt/eNOS pathway in the effect of U50,488H was studied using cultured endothelial cells subjected to artificial hyperlipidemia. Serum total cholesterol and low-density lipoprotein cholesterol concentrations dramatically increased after high-fat diet feeding. Administration of U50,488H significantly alleviated endothelial ultrastructural destruction and endothelium-dependent vasorelaxation impairment caused by hyperlipidemia. U50,488H also increased Akt/eNOS phosphorylation and serum/medium NO level both *in vivo* and *in vitro*. U50,488H increased eNOS activity and suppressed iNOS activity *in vivo*. The effects of U50,488H were abolished *in vitro* by siRNAs targeting κ-opioid receptor and Akt or PI3K/Akt/eNOS inhibitors. All effects of U50,488H were blocked by nor-BNI. These results demonstrate that κ-opioid receptor stimulation normalizes endothelial ultrastructure and function under hyperlipidemic condition. Its mechanism is related to the preservation of eNOS phosphorylation through activation of the PI3K/Akt signaling pathway and downregulation of iNOS expression/activity.

Endothelium is the major regulator of vascular homeostasis and plays a key role in physiology and pathology of the vascular system. It maintains the balance between vasodilation and vasoconstriction, thrombogenesis and fibrinolysis, inhibition and stimulation of smooth muscle cell proliferation as well as inflammation/anti-inflammation and oxidation/antioxidation. Cardiovascular risk factors alter endothelial function and trigger molecular events that disturb balances mentioned above^1^. Thus, endothelial dysfunction occurs, which is considered as an early event for atherosclerosis before angiographic or ultrasonic evidence^2^. Hyperlipidemia is an independent risk factor for many cardiovascular diseases. Excessive lipids in serum leads to accumulation and oxidation of low-density lipoprotein cholesterol (LDL-C) within the intima of the vessel wall^1^, causing endothelial dysfunction through inflammation, oxidation and eNOS uncoupling^3^,^4^, all of which are crucial steps leading to atherosclerosis. Therefore, approaches that maintain the function of the endothelium in hyperlipidemia hold great promise in preventing pathogenesis of atherosclerosis in early stage.

The maintenance of vascular integrity requires numerous endothelium-derived substances, among which nitric oxide (NO) is the most potent vasodilator. NO is formed in endothelial cells from its precursor L-arginine mainly by endothelial NO synthase (eNOS) in normal blood vessels. Under physiological conditions, NO dilates blood vessels, inhibits platelet aggregation and adhesion, and suppresses leukocyte infiltration. It also inhibits proliferation of vascular smooth muscle cells and oxidation of LDL^5^. However, elevation in serum lipids increases the production of reactive oxygen species (ROS), which reacts with NO to produce ONOO^−^ and causes eNOS uncoupling. ONOO^−^ can both directly damage eNOS and oxidize its cofactor BH_4_. Uncoupled eNOS also produces ONOO^−^. Combined with enhanced iNOS expression and activity it causes further damage to endothelium^4^. Theoretically, an approach that possesses functions of stimulating NO production and inhibiting ONOO^−^ formation would provide the best protection against vascular endothelial dysfunction^6^.

Our previous work demonstrated that κ-opioid receptor (κ-OR) stimulation with U50,488H directly dilates vessels in a NO-dependent manner^7^. It also attenuates pulmonary arterial pressure in rats with hypoxic pulmonary hypertension and effectively protects pulmonary artery endothelium through preservation of eNOS activity and anti-apoptotic effect^6^. κ-OR stimulation also showed anti-inflammatory effect in a rat model of diabetes^8^. Thus, the present study was designed to determine whether κ-OR stimulation with U50,488H protects endothelial function in hyperlipidemia and its underlying mechanisms.

## Results

### Effect of U50,488H on serum lipid profiles

**Figure 1:**
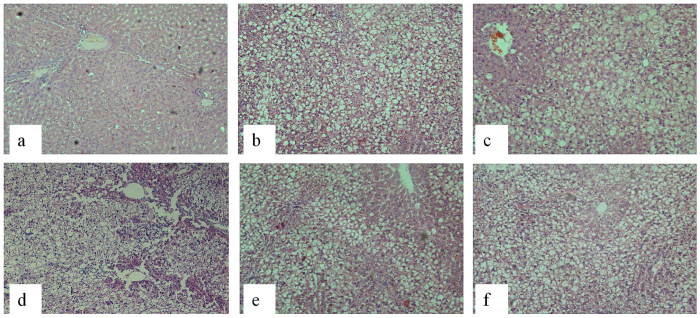
Representative H/E staining graphs of paraffin sections of the rat liver tissue (100×). (**a**) ND, (**b**) HFD, (**c**) HFD + V, (**d**) HFD + U, (**e**) HFD + N, (**f**) HFD + U + N. ND: normal diet group, HFD: high-fat diet group, HFD + V: high-fat diet + saline group, HFD + U: high-fat diet + U50,488H group, HFD + N: high-fat diet + nor-BNI group, HFD + U + N: high-fat diet + U50,488H + nor-BNI group. Our results showed apparent fatty degeneration in rat liver tissue after high-fat diet feeding. Neither U50,488H nor nor-BNI treatment improved the situation.

After 14 weeks, serum total cholesterol (TC) and LDL-C concentrations dramatically increased in groups fed with a high-fat diet (Table 1). However, body weight, fasting blood glucose, triglyceride (TG) and high-density lipoprotein cholesterol (HDL-C) concentrations did not change in these groups. Pretreatment with U50,488H and nor-BNI elicited no significant effect on these parameters. Apparent fatty degeneration in rat liver tissue after high-fat diet feeding was observed (Fig. 1). Neither U50,488H nor nor-BNI improved the fatty degeneration. These results demonstrate that a rat model of hyperlipidemia characterized by increased TC and LDL was successfully established.

### U50,488H alleviated hyperlipidemia-induced ultrastructural lesion of the aorta

**Figure 2:**
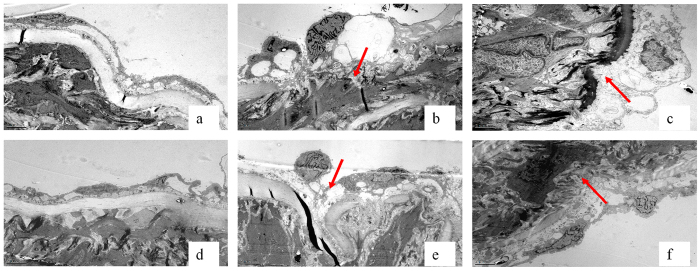
Representative transmission electron microscope graphs of ultrathin sections of the rat thoracic aorta (15,000×). (**a**) ND, (**b**) HFD, (**c**) HFD + V, (**d**) HFD + U, (**e**) HFD + N, (**f**) HFD + U + N. ND: normal diet group, HFD: high-fat diet group, HFD + V: high-fat diet + saline group, HFD + U: high-fat diet + U50,488H group, HFD + N: high-fat diet + nor-BNI group, HFD + U + N: high-fat diet + U50,488H + nor-BNI group. Arrows show discontinuous endothelial basement membrane and swollen endothelial cells sometimes with platelets adhered in impaired endothelium.

Aorta endothelial cells and internal elastic membrane presented with intact morphology in the ND group (Fig. 2). Abnormal ultrastructural changes of the aorta segments from the HFD and HFD+V groups were observed, which showed discontinuous endothelial basement membrane and swollen endothelial cells, sometimes with adhered platelets. U50,488H alleviated the lesion, turning the intima towards normal morphological pattern. The effect of U50,488H was blocked by nor-BNI.

### U50,488H preserved ACh-induced vasorelaxation in aorta segment from hyperlipidemic rats

**Figure 3:**
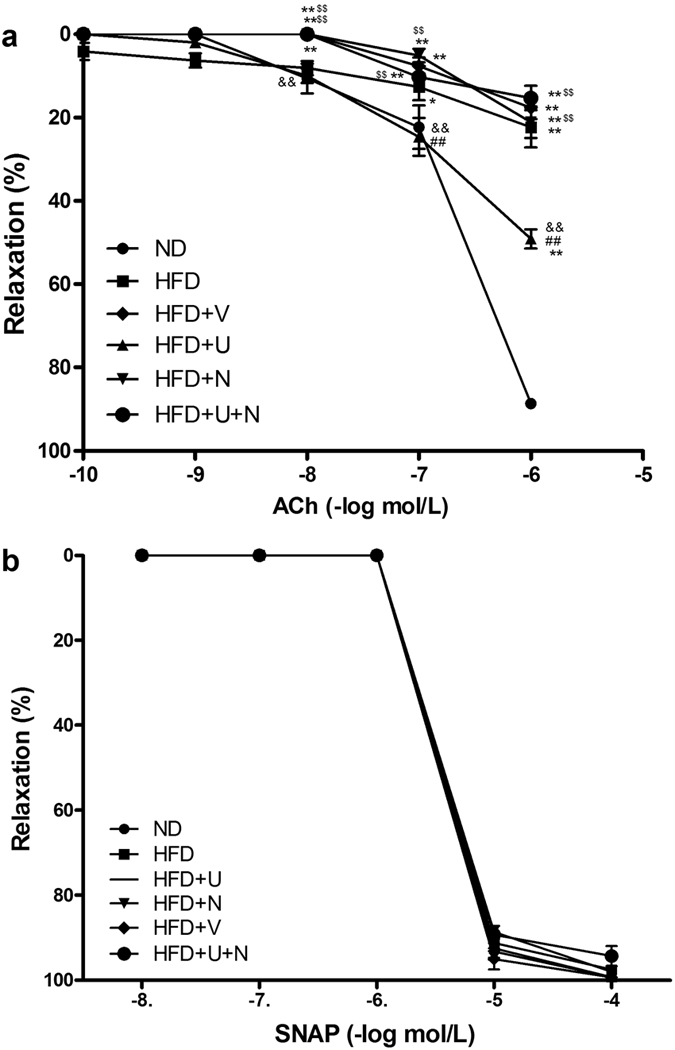
Concentration-dependent vasorelaxation of aortic segments in response to acetylcholine (Ach) (**a**) and S-nitroso-N-acetylpenicillamine (SNAP) (**b**) n = 8 segments/group from 10–12 rats. Values are mean ± SEM. ND: normal diet group, HFD: high-fat diet group, HFD + V: high-fat diet + saline group, HFD + U: high-fat diet + U50,488H group, HFD + N: high-fat diet + nor-BNI group, HFD + U + N: high-fat diet + U50,488H + nor-BNI group. *P < 0.05, **P < 0.01 vs. ND, ^##^*P* < 0.01 vs. HFD, ^&&^*P* < 0.01, vs. HFD + V, ^$$^*P* < 0.01, vs. HFD + U.

To clarify whether U50,488H protects endothelial function, isolated aorta rings (from aorta abdominalis) in different treatment groups were collected to conduct endothelium-dependent and -independent vasodilation as described. As shown in Fig. 3, compared with ND group, concentration-dependent vasorelaxation in response to acetylcholine (ACh) was impaired in all groups receiving a high-fat diet (Fig. 3a). However, vasorelaxation in response to the endothelium-independent vasodilator (S-nitroso-N-acetylpenicillamine, SNAP) was similar in all groups (Fig. 3b). These results indicated that hyperlipidemia caused significant endothelial dysfunction. Intriguingly, treatment with U50,488H exerted a protective effect on endothelial function, which was demonstrated by a significant improvement of vasorelaxation in response to ACh in HFD + U group compared with other groups receiving a high-fat diet. This effect was blocked by nor-BNI. Results above indicate that the protective effect of U50,488H is mediated by κ-OR.

### U50,488H increased the production of NO in hyperlipidemic rats and palmitate-treated HUVECs

**Figure 4:**
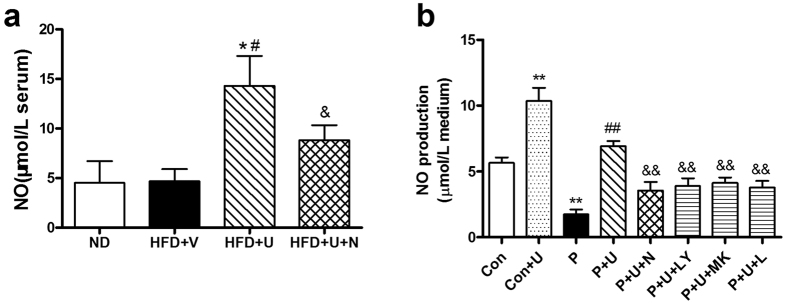
Effects of U50,488H on NO production in the absence and presence of nor-BNI and PI3K/Akt/eNOS inhibitor in serum (**a**) and culture medium for HUVECs (**b**) n = 10 (serum), n = 5 (cell culture medium). Values are mean ± SEM. ND: normal diet group, HFD: high-fat diet group, HFD + V: high-fat diet + saline group, HFD + U: high-fat diet + U50,488H group, HFD + N: high-fat diet + nor-BNI group, HFD + U + N: high-fat diet + U50,488H + nor-BNI group. Con: normal medium group, Con + U: normal medium + U50,488H group, P: palmitate-added medium group, P + U: palmitate-added medium + U50,488H group, P + U + N: palmitate-added medium + U50,488H + nor-BNI group, P + U + LY: palmitate-added medium + U50,488H + LY294002 group, P + U + MK: palmitate-added medium + U50,488H + MK2206-HCl group, P + U + L: palmitate-added medium + U50,488H + L-NAME group. *P < 0.05 vs. ND, ^#^*P* < 0.05 vs. HFD + V, ^&^*P* < 0.05 vs. HFD + U (**a**). **P < 0.01 vs. Con, ^##^*P* < 0.01 vs. P, ^&&^*P* < 0.01 vs. P + U (**b**).

To further investigate the mechanisms involved in the improvement of endothelial function, serum NO content was measured in all groups. As demonstrated in Fig. 4a, feeding with a high-fat diet for 14 weeks did not significantly alter serum NO content. Administration of U50,488H significantly elevated the serum NO level. The effect of U50,488H was abolished by nor-BNI. Palmitate treatment greatly decreased NO production in HUVECs. Treatment with U50,488H partly compensated this decrease. The effect of U50,488H was blocked not only by nor-BNI but also by LY294002, a PI3K inhibitor, MK-2206-2HCl, an Akt inhibitor, and L-NAME, an eNOS inhibitor, respectively in HUVECs (Fig. 4b). These results suggest that U50,488H increases NO production via a κ-OR-mediated PI3K/Akt/eNOS signaling pathway.

### U50,488H enhanced Akt/eNOS phosphorylation and eNOS activity but attenuated iNOS expression/activity in aorta of hyperlipidemic rats

**Figure 5:**
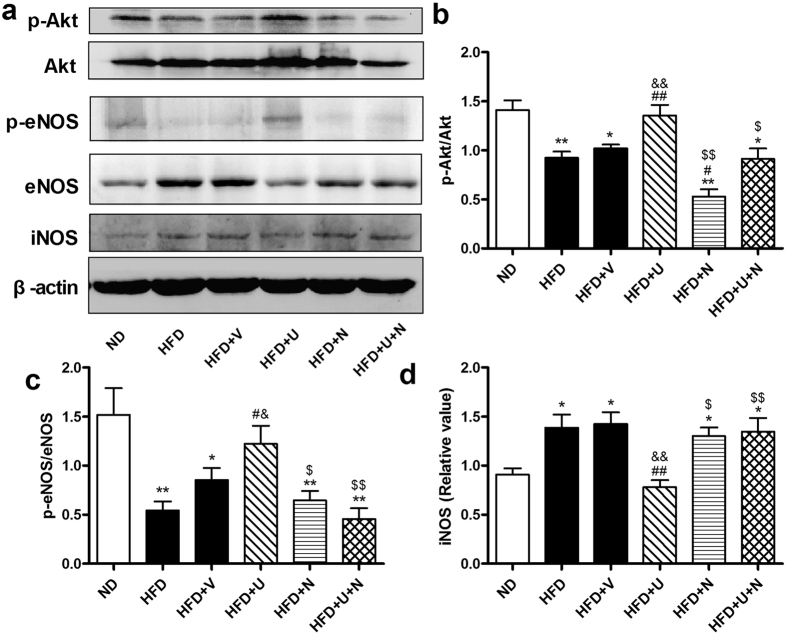
Effects of U50,488H on phosphorylation of Akt, eNOS and expression of iNOS in the absence and presence of nor-BNI *in vivo*. (**a**) Representative blots of total and phosphorylated Akt, eNOS and iNOS in aorta tissue. (**b**) Statistical data obtained from quantitative densitometry of Akt blots. (**c**) Statistical data obtained from quantitative densitometry of eNOS blots. (**d**) Statistical data obtained from quantitative densitometry of iNOS blots. n = 10. Values are mean ± SEM. ND: normal diet group, HFD: high-fat diet group, HFD + V: high-fat diet + saline group, HFD + U: high-fat diet + U50,488H group, HFD + N: high-fat diet + nor-BNI group, HFD + U + N: high-fat diet + U50,488H + nor-BNI group. *P < 0.05, **P < 0.01 vs. ND, ^#^*P* < 0.05, ^##^*P* < 0.01 vs. HFD, ^&^*P* < 0.05, ^&&^*P* < 0.01vs. HFD + V, ^$^*P* < 0.05, ^$$^*P* < 0.01 vs. HFD + U.

**Figure 6:**
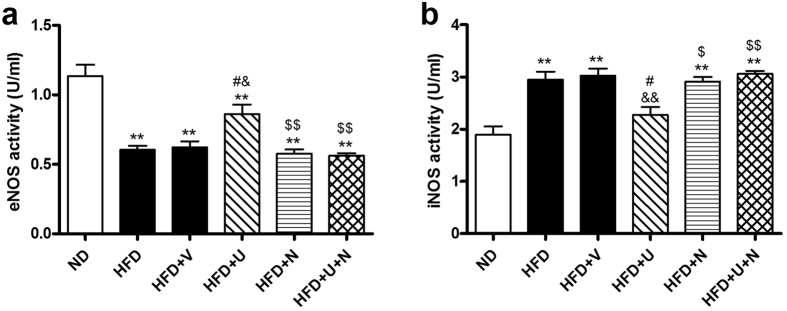
Effects of U50,488H on vascular tissue eNOS (**a**) and iNOS (**b**) activity in the absence and presence of nor-BNI (n = 10). Values are mean ± SEM. ND: normal diet group, HFD: high-fat diet group, HFD + V: high-fat diet + saline group, HFD + U: high-fat diet + U50,488H group, HFD + N: high-fat diet + nor-BNI group, HFD + U + N: high-fat diet + U50,488H + nor-BNI group. **P < 0.01 vs. ND, ^#^*P* < 0.05 vs. HFD, ^&^*P* < 0.05, ^&&^*P* < 0.01 vs. HFD + V, ^$^*P* < 0.05, ^$$^*P* < 0.01 vs. HFD + U.

We next investigated the change of Akt/eNOS pathway and iNOS in all groups. As shown in Fig. 5, hyperlipidemia significantly impaired Akt/eNOS phosphorylation, whereas U50,488H significantly restored Akt/eNOS phosphorylation. Interestingly, high-fat diet caused elevation of iNOS expression was significantly attenuated by administration of U50,488H (Fig. 5). As illustrated in Fig. 6, hyperlipidemia significantly decreased eNOS activity and elevated iNOS activity. Administration of U50,488H significantly restored eNOS activity and suppressed iNOS activity. All effects of U50,488H were abolished by nor-BNI. These data indicate that U50,488H possesses functions of both Akt/eNOS activation and iNOS suppression in a κ-OR-dependent manner.

### Effect of U50,488H was mediated by the PI3K/Akt/eNOS pathway

**Figure 7:**
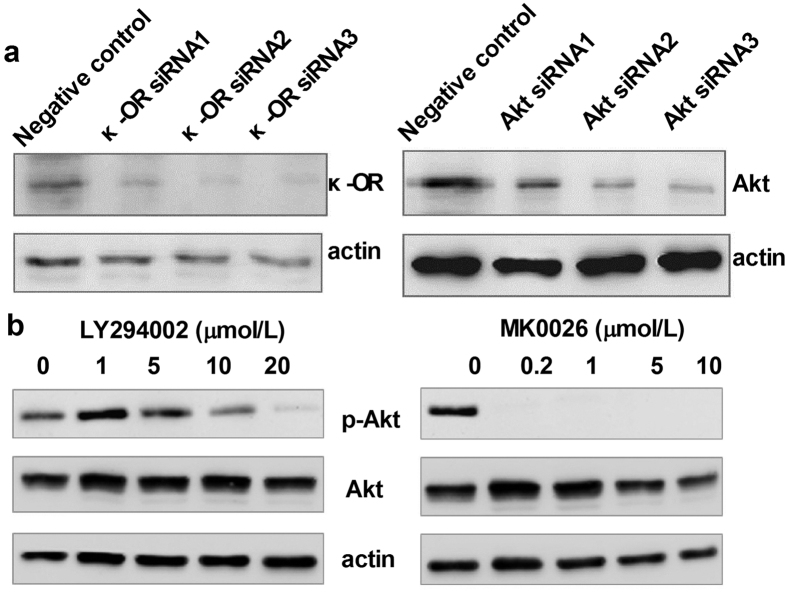
Test of the Akt/eNOS inhibitor (**a**) and siRNA targeting κ-OR and Akt (**b**) by Western blot.

**Figure 8:**
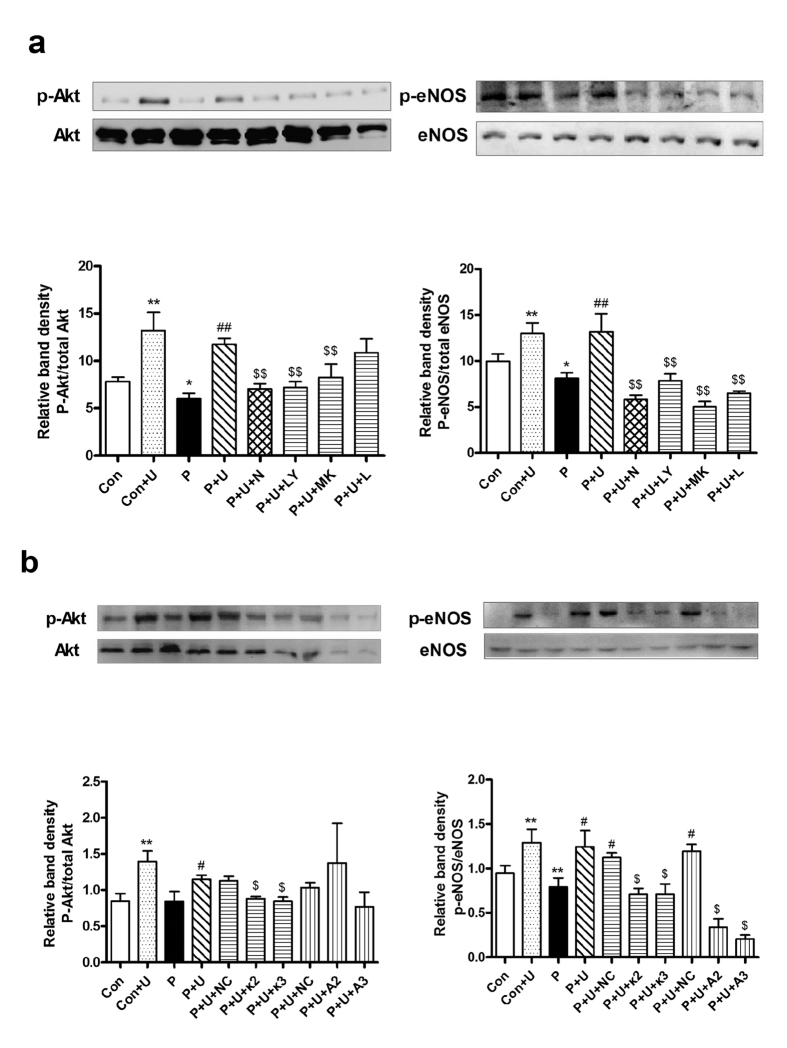
Effects of U50,488H on Akt/eNOS phosphorylation in the absence and presence of nor-BNI and PI3K/Akt/eNOS inhibitors (**a**) as well as siRNAs targeting κ-OR and Akt (**b**) *in vitro* (n = 5). Values are mean ± SEM. Con: normal medium group, Con + U: normal medium + U50,488H group, P: palmitate-added medium group, P + U: palmitate-added medium + U50,488H group, P + U + N: palmitate-added medium + U50,488H + nor-BNI group, P + U + LY: palmitate-added medium + U50,488H + LY294002 group, P + U + MK: palmitate-added medium + U50,488H + MK2206-HCl group, P + U + L: palmitate-added medium + U50,488H + L-NAME group. P + U + NC: palmitate-added medium + U50,488H + non-targeting siRNA group, P + U + κ2: palmitate-added medium + U50,488H + κ-OR siRNA2 group, P + U + κ3: palmitate-added medium + U50,488H + κ-OR siRNA3 group, P + U + A2: palmitate-added medium + U50,488H + Akt siRNA2 group, P + U + A3: palmitate-added medium + U50,488H + Akt siRNA3 group. *P < 0.05, **P < 0.01 vs. Con, ^#^*P* < 0.05 vs. P, ^##^*P* < 0.01 vs. P, ^$$^*P* < 0.01 vs. P + U (**a**), ^$^*P* < 0.05 vs. P + U + NC (**b**).

To confirm the role of the PI3K/Akt/eNOS pathway in the endothelial protective effect of U50,488H, we treated palmitate-stimulated HUVECs with siRNAs targeting κ-OR and Akt as well as chemical inhibitors of κ-OR (nor-BNI), PI3K (LY294002), Akt (MK-2206-2HCl) and eNOS (L-NAME). The effects of siRNAs and chemical inhibitors were tested by Western blot (Fig. 7). Although excessive palmitate in medium did not affect Akt/eNOS expression in HUVECs, it significantly reduced Akt/eNOS phosphorylation (Fig. 8a) and NO production (Fig. 4b). Preincubation of HUVECs with U50,488H restored the phosphorylation of Akt/eNOS (Fig. 8a) and NO production (Fig. 4b), which were abolished by nor-BNI, LY294002, MK-2206-2HCl and L-NAME (Fig. 8a). The protective effect of U50,488H was also blocked by siRNAs targeting κ-OR and Akt (Fig. 8b). These results suggest that κ-OR activation by U50,488H stimulates NO production in HUVECs exposed to artificial hyperlipidemia via the PI3K/Akt/eNOS signaling pathway.

## Discussion

As an independent risk factor of atherosclerosis, hyperlipidemia, especially hypercholesterolemia (as shown in our rat model), induces a series of molecular events including ox-LDL accumulation, eNOS uncoupling and iNOS upregulation, thus impairing endothelium^2^,^4^,^9^,^10^. Previous studies demonstrated that therapy has ability of activating the PI3K/Akt pathway, restoring eNOS activity and suppressing oxidation/nitration may be ideal solution to endothelial dysfunction^5^,^11^,^12^,^13^. In the present study we proved for the first time that preventive treatment with U50,488H showed a significant effect to ameliorate endothelial dysfunction in hyperlipidemia through activation of κ-OR and the PI3K/Akt/eNOS pathway. This conclusion is based on following observations: 1) Ultrastructure analysis proved that U50,488H has an endothelial protective effect from morphological aspect. 2) Relaxation response of aorta to ACh but not to SNAP was markedly improved by U50,488H. 3) U50,488H stimulated bioactive NO production, preserved eNOS activity and enhanced Akt/eNOS phosphorylation as well as inhibited iNOS expression. 4) The effect of U50,488H was via the PI3K/Akt/eNOS pathway as proved by chemical inhibition and RNAi in cultured HUVECs. 5) All the effects of U50,488H were abolished by nor-BNI. Our findings suggest that κ-OR plays an important role in the regulation of endothelial function. However, the protective effect of κ-OR stimulation is independent of serum lipid level since neither U50,488H nor nor-BNI altered serum lipid profiles or fatty degeneration in liver.

NO is a pivotal endothelium-derived protective substance. The production of NO and vascular response to it are sensitive indicators of vascular injury^2^. NO abnormality causes impaired endothelium-dependent vasodilation. The alteration in NO production and vascular response are responsible for the impired endothelium-dependent vasorelaxation in arteries of hyperlipidemic rats^2^,^5^,^14^. In our experiment, ACh-induced vasorelaxation in isolated aorta segment is endothelium and NO dependent, whereas SNAP can directly relax smooth muscle. Thus, the comparison between ACh and SNAP provides an ideal model for the evaluation of endothelial function. Our study showed that U50,488H treatment significantly preserved endothelium-dependent vasorelaxation in isolated aortic segments. At the same time, we found that serum/medium NO level were also increased by κ-OR stimulation. These effects were blocked by nor-BNI. Because ACh-induced vasorelaxation is NO dependent, we proved that the endothelial protective effect of κ-OR stimulation has a close connection with the preservation of NO.

The change of eNOS is an important mechanism in endothelium impairment caused by hyperlipidemia^2^,^4^,^14^,^15^,^16^. Evidence exists of reduced phosphorylation of eNOS in response to ox-LDL^17^. ox-LDL also alters the distribution of eNOS^2^. In some studies, eNOS expression was compensatorily enhanced^16^. Regardless of the manner in which its expression/phosphorylation changes, eNOS activity is inhibited in hyperlipidemia because it becomes uncoupled^2^. At the same time, as a response to hyperlipidemia and cytokines, iNOS expression is greatly upregulated^2^, which contributes to increased superoxide generation. In order to reveal the role of κ-OR in hyperlipidemia-induced NOS alteration, the expression/phosphorylation as well as activity of eNOS/iNOS were measured in our experiment. We demonstrated that serum NO level was not significantly changed in hyperlipidemic rats. This phenomenon may be attributed to the different alterations of eNOS and iNOS. We found that hyperlipidemia caused a significant decrease in Akt/eNOS phosphorylation and eNOS activity, but, at the same time, iNOS expression and activity increased greatly in aorta of hyperlipidemic rats. Perhaps NO produced by excessive iNOS compensated the loss from inactivated eNOS and stabilizes the total serum NO level. However, increased iNOS expression does not compensate the destructive effect of eNOS uncoupling and the imbalance between oxidation/antioxidation. Pretreatment with U50,488H restored Akt/eNOS phosphorylation and eNOS activity. It also suppressed iNOS expression/activity. The effect of U50,488H was blocked by nor-BNI. These results shows close relationship between κ-OR stimulation and Akt/eNOS pathway.

*In vitro*, we found a loss of NO production as well as Akt/eNOS phosphorylation in palmitate-treated HUVECs, which was restored by preventive treatment of U50,488H. Furthermore, the effect of U50,488H was significantly blocked by both siRNAs targeting κ-OR/Akt and chemical inhibitor to κ-OR (nor-BNI), PI3K (LY294002), Akt (MK-2206-2HCl) and eNOS (L-NAME). These results confirmed that the preservation of NO by U50,488H is mediated by the PI3K/Akt/eNOS pathway. This is also similar to our findings in chronic hypoxia-treated rats^6^.

In summary, our results achieved, for the first time, the most significant finding that κ-OR stimulation by U50,488H attenuates hyperlipidemia-induced endothelial dysfunction and ultrastructural lesion. We also found that U50,488H differentially regulates eNOS and iNOS activity in hyperlipidemic rats. This dual regulation of NOS by κ-OR stimulation is, to some extent, ideal for prevention of endothelial dysfunction. Our subsequent study using siRNAs and chemical inhibitors demonstrated that κ-OR stimulation by U50,488H exerts its effect through activation of the PI3K/Akt/eNOS signaling pathway. Our findings revealed the possibility that κ-OR used as a vascular protective drug target in clinical treatment of hyperlipidemia-induced cardiovascular diseases.

## Methods

### Animal model

Rat hyperlipidemia model was established as described previously^11^. Sixty male, 8-week-old Sprague Dawley rats obtained from the animal center of Fourth Military Medical University were randomly divided into six groups: normal diet group (ND), high-fat diet group (HFD), high-fat diet + saline group (HFD + V) (0.3 mL saline was intraperitoneally (i.p.) injected every 2 days), high-fat diet + U50,488H group (HFD + U) (1.25 mg/kg U50,488H was i.p. injected every other day^18^), high-fat diet + nor-BNI group (HFD + N) (2.0 mg/kg nor-BNI, a selective κ-OR antagonist, was i.p. injected every other day^18^), high-fat diet + U50,488H + nor-BNI group (HFD + U + N) (2.0 mg/kg nor-BNI was i.p. injected and 1.25 mg/kg U50,488H was i.p. injected 10 min later every other day^18^). ND group received a regular chow diet and all other groups received a high-fat (5% cholesterol supplemented) diet. Food and water were provided ad libitum. Animals were maintained in a temperature-controlled barrier facility with a 12:12 h light-dark cycle. U50,488H and nor-BNI were purchased from Tocris Bioscience (Bristol, UK). All experimental procedures were in accordance with the Guide for the Care and Use of Laboratory Animals published by the U.S. National Institutes of Health, NIH Publication No. 85–23 (revised 1996 and approved by the University Ethics Committee of the Fourth Military Medical University).

### Cell culture and treatment

The use of human umbilical vein endothelial cell lines (HUVECs) was reviewed and approved by the Ethics Committee of Fourth Military Medical University. HUVECs were purchased from ScienCell Research Laboratories (San Diego, CA). Cells were grown in EGM-2 BulletKit (CC-3162 Lonza) in a 5% CO_2_ incubator. Cells were used within passage 6 after primary culture. HUVECs were incubated with sodium palmitate (450 μmol/L) for 48 h to mimic hyperlipidemic condition^19^,^20^. Parallel groups were pretreated with PI3K inhibitors LY-294002 (20 μmol/L, Sigma), Akt inhibitor MK-2206-2HCl (1 μmol/L, Sigma), and eNOS inhibitor L-NAME (100 μmol/L, Sigma) following the manufacturers’ protocol. The siRNAs targeting human Akt (Genepharma) and human κ-OR (Genepharma) as well as control siRNA (nontargeting siRNA; Genepharma) were transfected to HUVECs using HiPerfect transfection reagent (Qiagen). HUVECs and culture medium were harvested at indicated times. Further examinations regarding the expression and phosphorylation of Akt/eNOS and NO production were done.

### Determination of serum lipid, glucose, serum/medium NO contentas well as H/E staining of paraffin sections of the rat liver tissue

After a high-fat diet feeding for 14 weeks, animals were weighed and anesthetized by i.p. administration of 10% chloral hydrate after fasting for 12 h. Blood was collected for determination of TC, TG, LDL-C, HDL-C and fasting blood glucose by a biochemistry analyzer (Cobas Integra 400 Plus, Roche) following the manufacturer’s protocol. Serum total nitric oxide content (NO_x_) was determined by measuring the concentration of nitrite, a stable metabolite of nitric oxide, through a modified Griess reaction method^11^. Briefly, serum was taken and mixed with an equal volume of modified Griess reagent. The Griess reagent consisted of 1% sulfanilamide, 0.1% N-(1-Naphthyl)ethylenediamine dihydrochloride, and 2% phosphoric acid. The concentration of the resultant chromophore was spectrophotometrically determined at 540 nm (Spectra Max Plus384, Molecular Devices). The same method was used to measure NO content in culture medium. Liver tissue was collected from rats and subjected to H/E staining to evaluate the fatty degeneration caused by high-fat feeding. Paraffin sections were made in the Department of Pathology, Xijing Hospital.

### Ultrastructural analysis of aorta

The aortic segment from the heart to theiliac bifurcation was excised and placed in ice-cold Krebs-Henseleit (K-H) buffer. K-H buffer consisted of (mmol/L) 118 NaCl, 4.75 KCl, 2.54 CaCl_2_·2H_2_O, 1.19 KH_2_PO_4_, 1.19 MgSO_4_·7H_2_O, 25 NaHCO_3_, 10.0 glucose, pH = 7.4. The aorta was cleaned and cut into 2-mm rings for both ultrastructure analysis and endothelial function assays. Fresh aortic segments from the same sites were dissected into sections (1 × 1 × 1 mm), placed into 4% precooled glutaraldehyde for fixation at room temperature for 6 h and then fixed in 1% osmium tetroxide for 2 h. Dehydration was completed by ethyl hydroxide and dimethyl ketone. Acetone-EPON812 epoxide resin was added for embedding, trimming, and slicing. After staining with uranyl acetate and lead citrate, intima and SMCs were observed under transmission electron microscope (JEM1400, HITACHI).

### Observation of endothelial function

Endothelial function was determined by comparing the vasorelaxation response to ACh, an endothelium-dependent vasodilator, with that of SNAP, an endothelium-independent vasodilator, as reported previously^6^. Briefly, aortic rings were mounted onto hooks, suspended in organ chambers filled with K-H buffer and aerated with 95% O_2_ and 5% CO_2_ at 37 °C, then connected to force transducers (WPI, Sarasota, FL) to record changes via Maclab data acquisition system. After equilibration for 60 min at a preload of 1 g, the rings were precontracted with norepinephrine (NE; 0.1 nmol/L). Once a stable contraction was achieved, the rings were exposed to cumulative concentrations of ACh (10^−10^ to 10^−6 ^mol/L). After the cumulative response stabilized, the rings were washed and allowed to equilibrate to baseline. The procedure was then repeated with an endothelium-independent vasodilator (SNAP, 10^−9^ to 10^−4 ^mol/L). Endothelial dysfunction was defined as a reduced vasorelaxation in response to ACh accompanied by an unchanged response to SNAP in one aorta ring.

### Western blot analysis of Akt, eNOS and iNOS expression/phosphorylation

Aortic segments and HUVECs were lysed in buffer (1 mM each: antipain, benzamidine, leupeptin, pepstatin A, and phenylmethylsulfonylfluoride (PMSF), 1% sodium dodecyl sulphate (SDS), and 5 mM ethylenediaminetetraacetic acid (EDTA)). Protein quantification was conducted with the BCA protein assay kit (Pierce, Thermo Fisher). Equal amounts of protein (40 μg protein/lane) were electrophoresed on a 10% SDS-polyacrylamide gel and electrophoretically transferred to a polyvinylidene difluoride membrane (Millipore, Billerica, MA). After blocking with 3% bovine serum albumin in Tris-buffered saline at room temperature for 1 h, the membranes were incubated with antibody against Akt/phosphorylated Akt (Cell Signaling Technology, Danvers, MA), eNOS/phosphorylated eNOS (BD Bioscience Laboratories, San Jose, CA) and iNOS (Millipore, Billerica, MA) overnight at 4 °C. The membranes were then washed with PBST and incubated with horseradish peroxidase-conjugated IgG antibody for 1 h at room temperature. β-Actin (Cell Signaling Technology) was selected as the loading control. Immunoblotting was detected using an enhanced chemiluminescence detection kit (Millipore) with ChemiDocXRS system (Bio-Rad Laboratory, Hercules, CA). The blot densities were analyzed with Quantity One Software (Bio-Rad Laboratory, Hercules, CA).

### eNOS and iNOS activity assays

Aortic tissues were homogenized in 0.9% NaCl solution (2 μl/μg) with a Heidolph DIA900 tissue homogenizer (Heidolph Instruments, Schwabach, Germany). The homogenate was centrifuged for 3000 rpm at 4 °C for 10 min to obtain the supernatant. Total NOS activity and iNOS activity were determined using an NOS activity assay kit (Nanjing Jiancheng Bioengineering Institute, Nanjing, China) following the manufacturer’s protocol as previously described^18^. eNOS activity was calculated by subtracting iNOS activity from the total NOS activity.

### Statistical analysis and artwork creation

Data were presented as mean ± SEM. All data were analyzed with either t-test (two groups) or ANOVA (three or more groups). After analysis by either t-test (two groups) or ANOVA, Bonferroni correction was used for post hoc t-tests. *P* < 0.05 was considered to be statistically significant. Graphpad Prism 5 was used to create all artworks.

## Additional Information

**How to cite this article**: Tian, F. *et al*. κ-Opioid Receptor Stimulation Improves Endothelial Function via Akt-stimulated NO Production in Hyperlipidemic Rats. *Sci. Rep.* **6**, 26807; doi: 10.1038/srep26807 (2016).

## Figures and Tables

**Table 1:** Serum glucose and lipid profiles.

Groups	ND	HFD	HFD + V	HFD + U	HFD + N	HFD + U + N
Glu mmol/l	9.17 ± 0.31	9.39 ± 0.54	8.56 ± 0.28	8.96 ± 0.25	9.06 ± 0.29	8.99 ± 0.45
TG mmol/l	1.23 ± 0.12	1.71 ± 0.17	1.16 ± 0.21	1.47 ± 0.15	1.42 ± 0.11	1.61 ± 0.17
TC mmol/l	1.50 ± 0.08	2.55 ± 0.18^**^	2.42 ± 0.36^*^	2.16 ± 0.08^*^	2.30 ± 0.19^**^	2.69 ± 0.22^**^
LDL-C mmol/l	0.16 ± 0.01	0.36 ± 0.02^**^	0.32 ± 0.05^*^	0.31 ± 0.03^**^	0.29 ± 0.04^**^	0.39 ± 0.02^**^
HDL-C mmol/l	0.40 ± 0.02	0.34 ± 0.02	0.35 ± 0.03	0.31 ± 0.02	0.32 ± 0.02	0.33 ± 0.02

Note: Mean ± SEM, n = 10.

ND: normal diet group, HFD: high-fat diet group, HFD + V: high-fat diet + saline group, HFD + U: high-fat diet + U50,488H group, HFD + N: high-fat diet + nor-BNI group, HFD + U + N: high-fat diet + U50,488H + nor-BNI group.

*P < 0.05, **P < 0.01 vs. ND group.
